# Process Optimization of SiC-Reinforced Aluminum Matrix Composites Prepared Using Laser Powder Bed Fusion and the Effect of Particle Morphology on Performance

**DOI:** 10.3390/ma17051187

**Published:** 2024-03-04

**Authors:** Xinghua Ji, Shufeng Li, Huiying Liu, Xin Li, Xin Zhang, Lei Liu, Shaolong Li, Lina Gao, Shaodi Wang, Biao Chen, Yuanbao Li

**Affiliations:** 1 School of Materials Science and Engineering, Xi’an University of Technology, No. 5 Jinhua South Road, Xi’an 710048, China; jxhlucky@163.com (X.J.); 1220112030@stu.xaut.edu.cn (H.L.); lx1998952@163.com (X.L.); mrliu_lei@126.com (L.L.); li1046380495@163.com (S.L.); lngao001@163.com (L.G.); shaodiwang2023@163.com (S.W.); 2 Shandong Province Key Laboratory of Powder Metallurgy in Advanced Manufacturing, Laiwu Vocational and Technical College, No. 1 Shancai Street, Laiwu 271100, China; 13002769728@163.com; 3 Centre of Excellence for Advanced Materials, Songshan Lake, Dongguan 523808, China; 4 Chongqing Innovation Center, Northwestern Polytechnical University, Chongqing 401120, China; chen@nwpu.edu.cn

**Keywords:** aluminum matrix composites, process optimization, laser powder bed fusion, SiC particle, mechanical properties

## Abstract

Process parameters and powder spreading quality are important factors for aluminum matrix composites (AMCs) prepared using laser powder bed fusion (LPBF). In this study, a Box–Behnken Design (BBD) was used to optimize the process parameters, and near-spherical β-SiC was selected to improve the quality of powder spreading. The rationality of parameter optimization was verified by testing the density of samples prepared using different laser power levels. Al_4_C_3_ diffraction peaks were found in XRD patterns, which indicated that interface reactions occurred to form good interface bonding between the Al matrix and the SiC particles. The tensile strength and plasticity of LPBF α-SiC/AlSi10Mg were lower than that of LPBF AlSi10Mg, which was mainly due to the poor fluidity of the powder mixtures and powder spreading quality. For LPBF β-SiC/AlSi10Mg, the tensile strength increased and elongation decreased slightly compared to LPBF α-SiC/AlSi10Mg. The data in this study were compared with the data in other studies. In this study, LPBF AlSi10Mg and LPBF β-SiC/AlSi10Mg not only showed the inherent high strength of their LPBF parts, but also had relatively high plasticity. Matching between strength and plasticity was mainly dependent on the scanning strategy. Most studies use uni-directional or bi-directional scanning strategies with a certain rotation angle between layers. A chessboard scanning strategy was used in this study to form a coarse remelted connected skeleton inside the material and significantly improve plasticity. This study lays a theoretical and experimental foundation for the controllable preparation of SiC-reinforced AMCs using LPBF.

## 1. Introduction

SiC particle (SiCp)-reinforced aluminum matrix composites (AMCs) have been widely used in aerospace, transportation, and other fields due to advantages such as high specific strength and specific modulus and good wear resistance and thermal stability [1,2,3,4]. However, SiCp-reinforced AMCs prepared by conventional processes, such as casting [5], infiltration [6], or powder metallurgy [7], frequently exhibit weaknesses such as coarse precipitates, inadequate interface bonding between the SiCps and the aluminum matrix, and difficulty in producing complex structural components directly. These bottleneck problems often limit the AMCs’ performance improvements [8,9]. Considering the growing demand for high-performance components with complex structures in modern equipment systems, traditional processes can no longer meet the application needs of SiCp-reinforced AMCs [10,11]. Laser powder bed fusion (LPBF) is one of the most well-established additive manufacturing (AM) methods due to its use of non-equilibrium melting and rapid solidification, which can significantly refine microstructures and improve the interface bonding between the reinforcements and the matrix. Its process parameters are flexible and adjustable, which can regulate interface reactions during LPBF [12,13]. Therefore, LPBF holds significant potential for developing and applying parts with a high load-bearing capacity, exceptional precision, and intricate complexity [14,15,16].

AlSi10Mg, a hypoeutectic cast aluminum alloy, is developed for LPBF based on its composition and melting characteristics. Si can enhance the laser absorption rate of the alloy powder, which can lead to a reduction in laser power requirements, an increase in the molten pool temperature, and an improvement in the melt fluidity. The addition of Si can also improve the flowability of the powder and reduce the shrinkage rate and hot cracking tendency. The rapid solidification process can control the morphology and size of Si, which can improve the properties of the alloy. Hence, AlSi10Mg demonstrates significant potential for widespread application in the realm of high-performance components with complex structures. Li [17] employed a strip exposure strategy to fabricate AlSi10Mg alloy using various laser energy densities, and they revealed that laser energy density values within the range of 77.8–144 J/mm^3^ had minimal influence on the relative density of the components. Moreover, it was observed that lower laser energy densities resulted in smaller grain sizes compared to higher laser energy densities. Using in situ compression testing, Wu [18] analyzed the changes in the dislocation structure of AlSi10Mg prepared with LPBF. The results showed that the cell structure boundaries and particles within the structure limited dislocation movement during the deformation process. Lu [19] conducted an analysis of the variations in microstructure gradient and element distribution arising from the cooling speed of the molten pool during LPBF. The differences in pool cooling speed played an important role in shaping the distribution of Si particles, dendrite size, and sub-boundary and sub-grain structures. Luca [20] investigated the influence of temperature, solution time, and aging treatment on the structure, hardness, and density of AlSi10Mg alloy manufactured through direct metal laser sintering. They compared these results with samples obtained through gravity casting and determined suitable heat treatment process parameters. In order to improve the performance of LPBF AlSi10Mg, its process parameters and defect formation mechanisms have been widely studied. Chu [21] confirmed that coarser powders are much more sensitive to changes in laser power in terms of melt pool characteristics, defect population, and size, showing significant improvements in relative density upon increasing laser power. Kumar [22] obtained optimal process parameters such as laser power, scanning speed, layer thickness, and hatch spacing, improving the structural integrity of the printed AlSi10Mg parts. Chen [23] proposed a novel approach in order to reduce defects, refine grains, and enhance hardness during the LPBF process, utilizing low laser power and a continuous scanning strategy. Wang [24] fabricated composites consisting of 2 wt.% nano-SiC and AlSi10Mg through a combination of ball milling and LPBF processes. Their research revealed that the inclusion of nano-SiC led to the grain refinement of the matrix and altered grain orientation. Nonetheless, the agglomeration of nano-SiC was inevitable, which prevented the performance of the nano-SiC-AlSi10Mg from being significantly enhanced [25]. Zhang [26] employed the LPBF process to produce 10 wt.% micron-SiC-AlSi10Mg composite materials at various laser energy densities. Their research revealed that laser energy density exerted a notable influence on density, grain size, and microstructure. SiC dispersed uniformly within the matrix and higher laser energy densities significantly promoted the in situ formation of Al_4_SiC_4_. Zou [27] investigated the influence of micron-SiC content on the density, microstructure, and mechanical properties of SiCp/AlSi7Mg manufactured using LPBF. Their research indicated that the relative density of the composite material initially rose and then declined as the SiC content increased, and the highest relative density of 99.20% was reached when the SiC content reached 5 wt.%. During the LPBF process, SiC particles react with the Al matrix to form the Al_4_SiC_4_ phase. The microhardness and tensile strength of SiCp/AlSi7Mg composites reached 148.40 HV and 451.89 MPa, which exhibited a notable increase compared to the AlSi7Mg alloy. However, the elongation rate of the composite was significantly lower than that of the AlSi7Mg alloy. Many scholars have also conducted research on surface smoothness and integrity to improve the practical application of LPBF components, and surface mechanical attrition treatment (SMAT) can improve the surface finish [28,29,30].

In this work, α-SiC is predominantly utilized to prepare SiCp-AMCs using LPBF; its irregular shape with sharp edges and corners can indeed pose challenges to the flow characteristics and powder dispersion quality of the mixed powder, which exerts a significant influence on the performance of the specimens [31,32]. Simultaneously, it has been observed that, even for the same materials, the optimal preparation process parameters can vary considerably in different studies. The selection of process parameters often relies on orthogonal experiments to determine the optimal combination of parameters. However, this does not establish a mathematical model to explicitly correlate these parameters with experimental outcomes. To assess the quality of the LPBF specimens, this study uses the Box–Behnken Design (BBD) of the response surface methodology (RSM). Multiple quadratic regression equations are employed to model the functional relationship between the parameters and the relative densities, and the optimal parameters are obtained by solving the regression equation. Meanwhile, β-SiC with a spherical-like shape was used as a reinforcement to analyze the impact of the morphology of the SiCps on the formability and performance of 2 wt.% SiC/AlSi10Mg prepared under the optimized parameters. The influence of the process parameters on the composite microstructure, mechanical properties, and interface bonding was systematically analyzed. This study lays a theoretical and experimental foundation for fabricating SiCp-reinforced AMCs with high performance.

## 2. Materials and Methods

### 2.1. Materials and Processes

**Figure 1:**
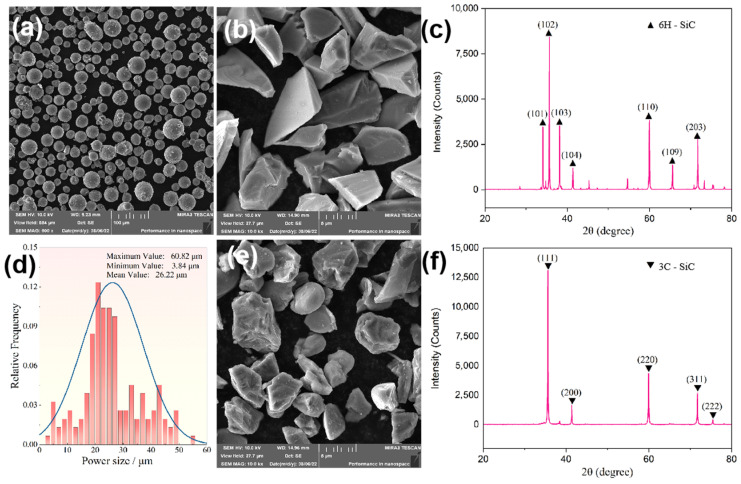
SEM images and X-ray diffraction patterns of the original powders: (**a**) AlSi10Mg; (**b**) α-SiC; (**c**) XRD pattern of α-SiC; (**d**) particle size distribution of AlSi10Mg powder; (**e**) β-SiC; (**f**) XRD pattern of β-SiC.

Commercial aerosolized AlSi10Mg powder (diameter: 15–53 μm) was used in this study. Its chemical composition is shown in Table 1. α-SiC and β-SiC were added for reinforcement, synthesized using a solid-phase synthesis method with an average particle size of 5 μm and a mass fraction of 2 wt.%. A micrograph and the phase composition of the original powder are shown in Figure 1. As shown in Figure 1a, AlSi10Mg powder particles exhibit high sphericity, smooth surfaces, and an average diameter of around 26 μm. The particle size distribution is illustrated in Figure 1d. The morphology of α-SiC is characterized by irregular shapes and sharp corners, as shown in Figure 1b. β-SiC particles exhibit superior natural sphericity without sharp edges along the surface, and their morphology is shown in Figure 1e. Compared to α-SiC, β-SiC has excellent thermal conductivity and a low coefficient of thermal expansion due to its significantly higher electrical conductivity, which results in minimal thermal stress during the heating and cooling processes. The X-ray diffraction (XRD) patterns of α-SiC and β-SiC are presented in Figure 1c,f, respectively.

The LPBF fabrication of SiCp/AlSi10Mg involved two steps: powder mixing and printing. First, powder mixing was carried out using planetary ball milling (PBM, PM-100, Retsch, Germany) at a rotation speed of 200 rpm and a ball-to-powder weight ratio of 5:1. Anhydrous ethanol was added to reduce the damage to the sphericity of the powder during the ball milling process. Then, the powder mixture was thoroughly dried at 80 °C for 10 h in a vacuum drying oven. Secondly, SiC/AlSi10Mg was prepared with an SLM150D printer (SLM150D, Tuobao, Wuhu, China); its detailed parameters are listed in Table 2. Vacuum pumping and argon gas injection were used to control oxidation during the printing process, and the oxygen content in the chamber was always controlled below 200 PPm. A chessboard scanning strategy was adopted to reduce the microstructure’s heterogeneity. The size of the chessboard was 4 mm, and the overlap between the chessboards was 0.1 mm. The laser scanning directions between adjacent chessboards were perpendicular to each other, with a 67° rotation and a 1 mm offset between each layer of a chessboard. The substrate did not undergo preheating treatment before printing, and the LPBF specimens were not subjected to any other heat treatment.

### 2.2. Material Characterization

**Figure 2:**
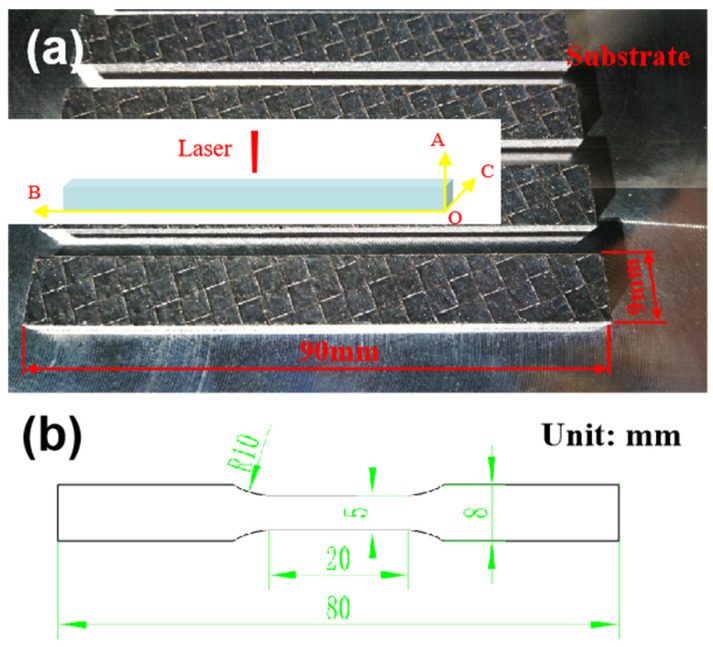
LPBF specimens and tensile specimen dimensions diagram: (**a**) LPBF specimens; (**b**) tensile specimen dimensions.

Phase analysis was carried out using X-ray diffraction (XRD-7000, Shimadzu, Kyoto, Japan) with a Cu Ka radiation source to identify the phases. The scanning was conducted at a voltage and current of 30 kV and 30 mA, respectively. Microstructure and morphology were observed using three different microscopes: an optical microscope (OLYMPUS GX71 type, referred to as OM), an ultra-depth three-dimensional microscope (AXIO, ZEISS, Oberkochen, Germany), and a field emission scanning electron microscope (JEOL JSM-6700, referred to as SEM). The specimen’s density was determined using the Archimedes drainage method. To assess the tensile properties of the materials, an HT-2402 universal tensile testing machine (HT-2402, HUANG HE, Zhengzhou, China) was employed. The specimens were printed to sizes of 90 mm × 9 mm × 5 mm as shown in Figure 2a, and the coordinate system of the printed specimen is established. Point O is located on the substrate, OA is the building direction, OB is the length direction, and OC is the width direction. The dimensions of the tensile specimens after cutting are depicted in Figure 2b. The tensile process was conducted with a constant tensile rate of 0.6 mm/min. For each set of samples, three specimens were tested and the average value was calculated to ensure the reliability of the dates.

## 3. Results

### 3.1. Process Parameter Optimization and Verification

During the LPBF process, the laser–powder interaction and laser energy input significantly impact the quality of the specimens. The primary process parameters influencing laser energy density include laser power (P), scanning speed (V), scanning spacing (D), and layer thickness (t). In this study, a layer thickness of 30 μm was used. The LPBF process parameters from references [33,34,35,36] were used for comparison. The parameter variables and levels are detailed in Table 3.

In this study, the software Design Expert v.12 was used for experimental design. The relative density (RD) was used as the response value to obtain the optimal parameters. Formula (1) is the relationship equation obtained by Design Expert v.12.

RD = 55.81745 + 0.268841 × P + 0.005849 × S − 0.034708 × D + 0.000025 × SD − 0.000415 × P^2^ − 0.0000027 × S^2^ (1)

The optimized parameters were P = 283 W, V = 2297 mm/s, and D = 58 μm, and the corresponding theoretical relative density is 99.05%. By comparing the coefficient values, it can be seen that P has the greatest impact on relative density. To validate the optimized process parameters, the laser power was changed while keeping all other parameters constant. Laser power levels of 200 W and 350 W were chosen to fabricate β-SiCp/AlSi10Mg specimens to confirm the accuracy of the parameter optimization.

**Figure 3:**
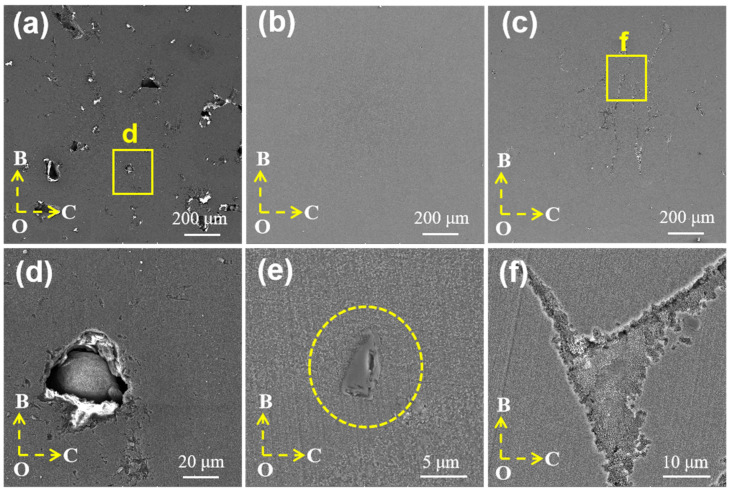
Microstructure of β-SiC/AlSi10Mg prepared under different laser powers: (**a**,**d**) 200 W; (**b**,**e**) 283 W; (**c**,**f**) 350 W.

**Figure 4:**
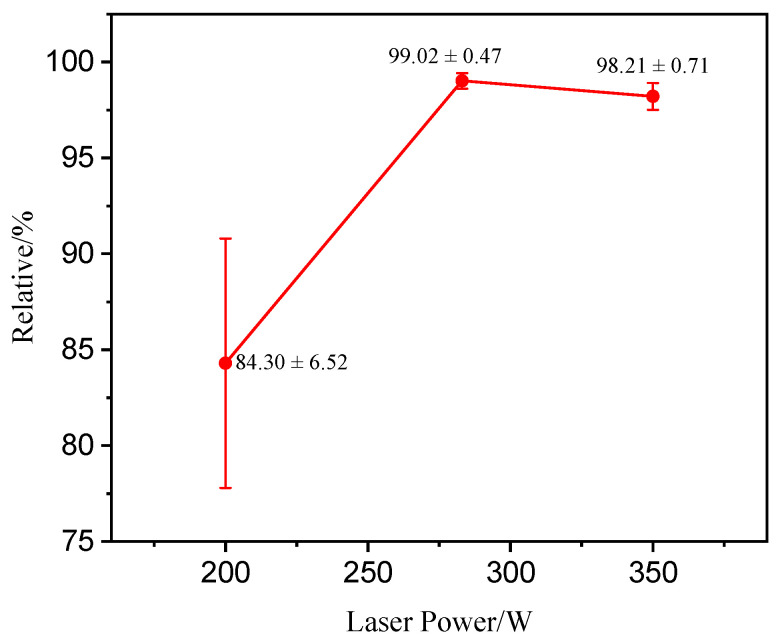
Relative density under different laser powers.

Figure 3 shows the microstructure of β-SiCp/AlSi10Mg prepared under different laser power levels. As shown in Figure 3a, the microstructure contains a higher number of pores, including large irregular pores and nearly round small pores, which can be attributed to the high viscosity and limited fluidity of the melt within the molten pool when the laser power is 200 W. Additionally, some powder particles do not melt due to low laser energy, as shown in Figure 3d. At the optimized process parameter of P = 283 W, the specimen exhibits good compactness, and the interface between SiC and the matrix is well bonded as shown in Figure 3b,e. When the power is increased to 350 W as shown in Figure 3c,f, the boundary of the molten pool becomes distinctly visible, and cracks and defects appear, which is related to the formation of pores during the LPBF process. Metal gasification intensifies with the increase in laser energy, and the bubble experiences pressure-driven growth, steam condensation, and diffusion, and then interacts with solidification microstructures, such as honeycomb dendrites, and can ultimately be captured by the advancing solidification front [37]. The rationality of this optimization method can be proven by comparing the densities of printed specimens at various laser powers (as depicted in Figure 4). At lower laser powers, the parts exhibit lower relative density, and there is a significant fluctuation in the density of the specimens, indicating molding instability. When the laser energy is increased to 350 W, the density reaches 98.21%, but it remains lower than the sample fabricated at 283 W, indicating that both density and molding stability are compromised at this higher power level.

### 3.2. Phase Analysis

**Figure 5:**
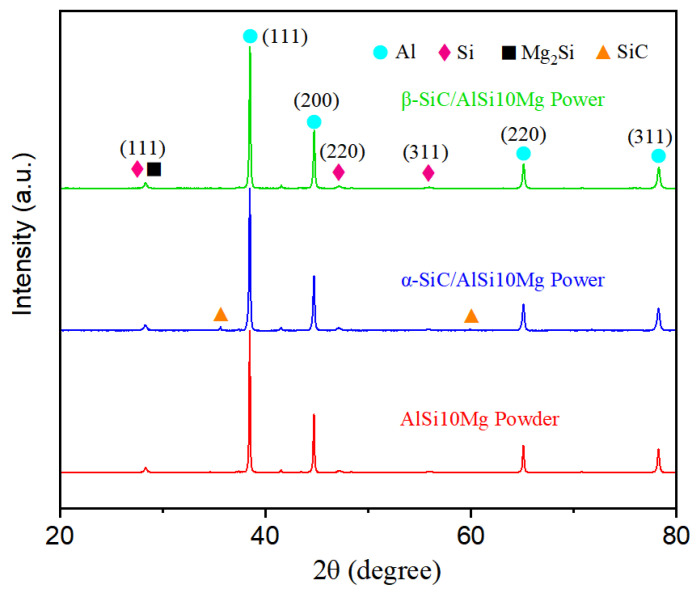
XRD patterns of AlSi10Mg powder, α-SiC/AlSi10Mg mixed powder, and β-SiC/AlSi10Mg mixed powder.

Figure 5 shows the X-ray diffraction (XRD) patterns of the original powders. It can be seen that the phase compositions of the matrix powder and mixed powder all contain Al, Si, and Mg_2_Si phases. Weak diffraction peaks of α-SiC were detected at 36° and 61°; however, no diffraction peaks of β-SiC were detected in the β-SiC/AlSi10Mg mixed powder. This difference may be due to insufficient addition or experimental errors.

**Figure 6:**
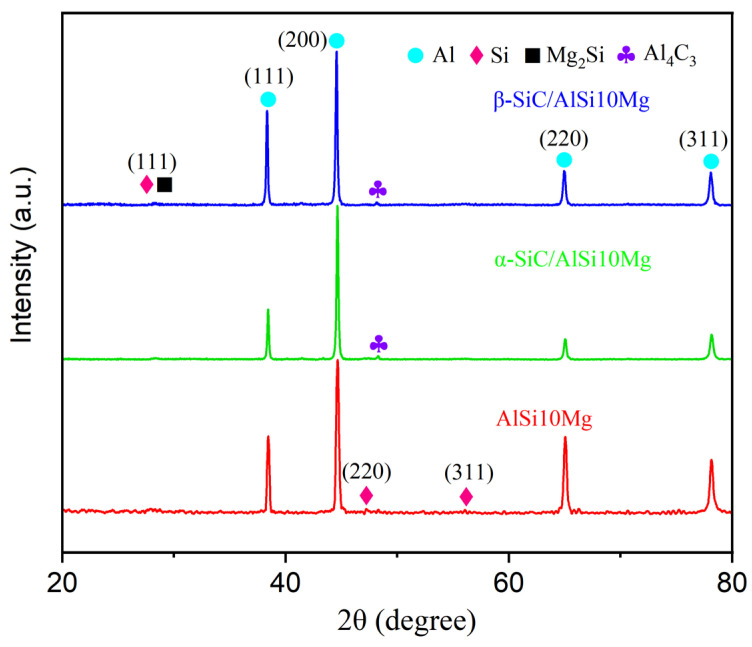
XRD patterns of LPBF AlSi10Mg, α-SiC/AlSi10Mg, and β-SiC/AlSi10Mg composites.

Figure 6 shows the XRD patterns of the AlSi10Mg, α-SiC/AlSi10Mg, and β-SiC/AlSi10Mg composites prepared by LPBF. It was observed that the composite specimen still contained Al, Si, and Mg_2_Si phases. However, the most significant change was observed in the Al phase, where the strongest peak shifted from (111) in the original powder to (200). This shift indicates that the LPBF specimen developed a noticeable texture during the rapid solidification process. SiC was not detected in the LPBF specimens, likely due to the low content. Furthermore, weak Al_4_C_3_ diffraction peaks were observed in SiC/AlSi10Mg composites. This suggests that SiC undergoes an interfacial reaction with the aluminum matrix during the LPBF process. The presence of trace interfacial reactant contributes to improving interfacial wetting and bonding between the matrix and the reinforcing phase, thereby enhancing load transfer efficiency.

### 3.3. Microstructure

**Figure 7:**
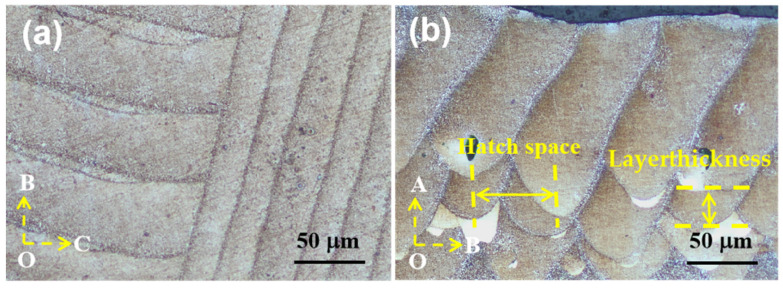
Metallographic photos of different observation surfaces of LPBF β-SiC/AlSi10Mg specimen: (**a**) BOC surface; (**b**) AOC surface.

Figure 7 shows the microstructure of the LPBF β-SiC/AlSi10Mg specimen. As shown in Figure 7a, the scanning surface reveals a distinctive scanning pattern with a 90° rotation between adjacent chessboards, and the molten pool boundaries are well defined. At the same time, it should be noted that the width of the molten pool was not equal, which should be equal according to the LPBF process. This is because the observation surface was not completely parallel to the scanning surface but was at a certain angle. Figure 7b shows the surfaces parallel to the scanning direction and the molten pool exhibiting a fish-scale-like pattern, which corresponds to the Gaussian energy distribution with higher energy density at its center and lower density at its edges. Consequently, the center of the laser beam created a deeper remelting effect compared to the scanning area at the edge of the beam, and so a unique fish-scale-like morphology was formed. The hatch spacing and the thickness of the printing layer were measured; the powder spreading thickness was set to 30 μm and kept constant, but the printing layer thickness was uneven. It can be inferred that the LPBF process is unstable because of unstable laser energy output, uneven powder distribution, wind speed, and other factors. Controlling the stability of the LPBF process is of great significance for improving the quality of LPBF parts.

**Figure 8:**
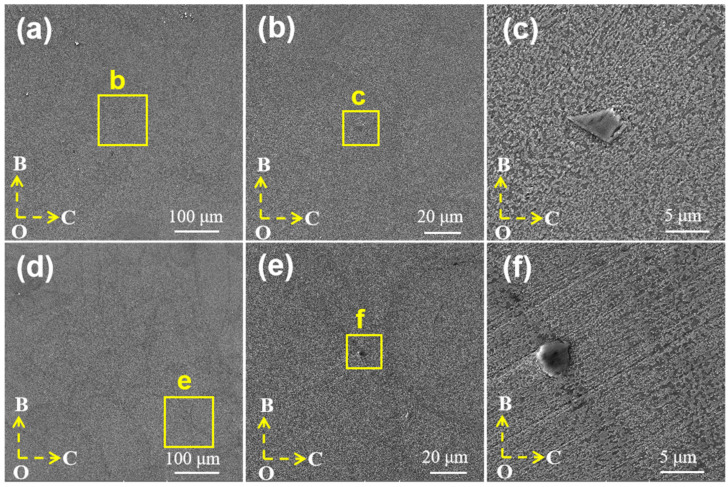
LPBF SiC/AlSi10Mg microstructure: (**a**–**c**) α-SiC/AlSi10Mg; (**d**–**f**) β-SiC/AlSi10Mg.

**Figure 9:**
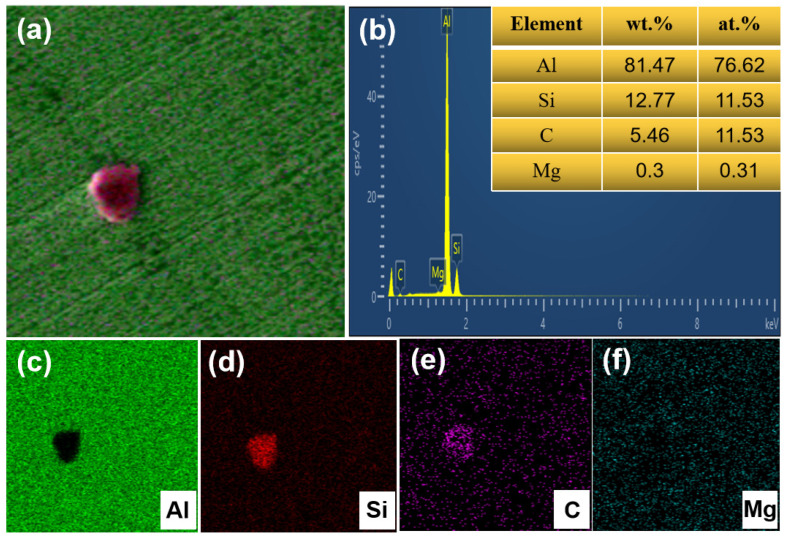
Energy spectrum analysis of LPBF β-SiC/AlSi10Mg: (**a**) element surface scan; (**b**) element proportion results; (**c**–**f**) Al, Si, C, Mg element surface distribution.

Figure 8 shows the microstructure of the LPBF α-SiC/AlSi10Mg and β-SiC/AlSi10Mg specimens. The boundaries of the molten pool in the composite material are distinctly visible as shown in Figure 8a,d, and SiC particles were detected at the boundaries of the molten pool that had bonded well with the matrix as shown in Figure 8b,e. In LPBF α-SiC/AlSi10Mg, as shown in Figure 8c, the reinforcement particles exhibit a noticeable irregular morphology, while they present a near-spherical shape in LPBF β-SiC/AlSi10Mg as shown in Figure 8f. An energy spectrum scan was employed to further determine the reinforcing phase, as shown in Figure 9. The Si and C atomic ratio of the reinforcing phase was 1:1 according to the element proportion results as shown in Figure 9b, which confirms that the reinforcing phase was β-SiC combining the results of the energy spectrum as shown in Figure 9c–f. The size of the reinforcing phase is approximately 5 μm, similar to the original β-SiC particle. By comparing Figure 8c,f, it is found that cracks appear at the sharp corners of α-SiC, which means that irregular SiC particles face challenges in rolling or rotating during the LPBF molding process. Consequently, this can affect the fluidity of the mixed powders and result in uneven powder spreading and incomplete filling of the powder layers. These factors, in turn, have an impact on the densities of the specimens and give rise to interfacial defects in the composites. In the β-SiC/AlSi10Mg composite specimen, a well-combined interface is observed between the β-SiC particles and the matrix with no evident defects. Through an analysis of SiC positioning and interfacial bonding, it becomes evident that during the melting and rapid solidification process, SiC is driven toward the molten pool boundary. The migration occurs because of the stirring effect induced by the Marangoni flow and the advancement of the solidification front.

### 3.4. Mechanical Properties

**Figure 10:**
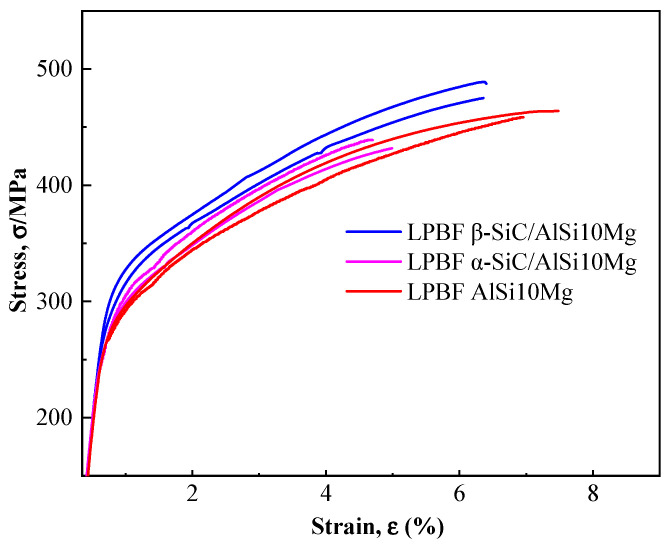
Tensile stress–strain curves of LPBF AlSi10Mg, α-SiC/AlSi10Mg, and β-SiC/AlSi10Mg specimens.

Figure 10 displays the tensile stress–strain curves of the LPBF AlSi10Mg, α-SiC/AlSi10Mg, and β-SiC/AlSi10Mg specimens. When P = 283 W, V = 2297 mm/s, and D = 58 μm, AlSi10Mg exhibits excellent plasticity and high tensile strength, with an elongation of 7.38% and tensile strength of 463 MPa. These excellent mechanical properties once again prove that the parameter optimization method used is reasonable. The LPBF α-SiC/AlSi10Mg specimen shows a decrease in both tensile strength and elongation, mainly due to the irregular shape of α-SiC, which leads to poor powder flowability and poor powder spreading quality, while for LPBF β-SiC/AlSi10Mg, the tensile strength increases and the elongation decreases slightly. The yield strength of SiC/AlSi10Mg is higher than that of AlSi10Mg, and the yield strength of β-SiC/AlSi10Mg increases more significantly compared with α-SiC/AlSi10Mg. The strengthening mechanisms of the LPBF β-SiC/AlSi10Mg specimen are as follows. Firstly, the addition of β-SiC increases the absorption of the laser during the LPBF process, so the melt viscosity decreases and the bonding between adjacent molten pools is strengthened [27]. The second is that the generation of Al_4_C_3_ indicates the metallurgical reaction between SiC and the Al matrix, which is conducive to the formation of a good bonding interface. As the reinforced phase, SiC directly bears the load transferred from the Al matrix, thus improving the strength of the composites. Furthermore, the mismatch of the thermal expansion coefficient between the Al matrix and SiC particles leads to the formation of a great number of geometric dislocations within the matrix, which can improve the strength of composite specimens [33,38]. SiC particles can improve the slip resistance of dislocations and enhance the strength of the composites. At the same time, due to the increase in porosity and the cutting effect of SiC on the matrix, the plasticity of LPBF β-SiC/AlSi10Mg shows a certain degree of decrease.

### 3.5. Fracture Mechanisms

**Figure 11:**
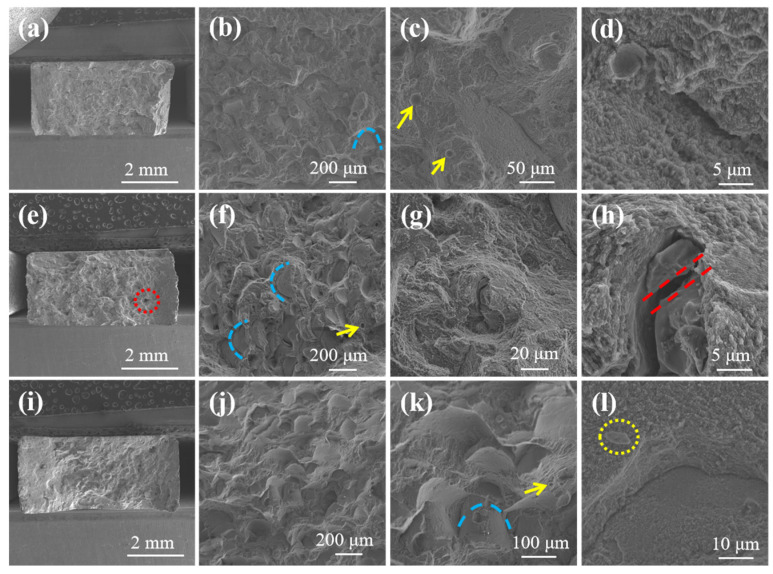
Fracture surface morphology of LPBF specimens: (**a**–**d**) AlSi10Mg alloy; (**e**–**h**) α-SiC/AlSi10Mg; (**i**–**l**) β-SiC/AlSi10Mg.

**Figure 12:**
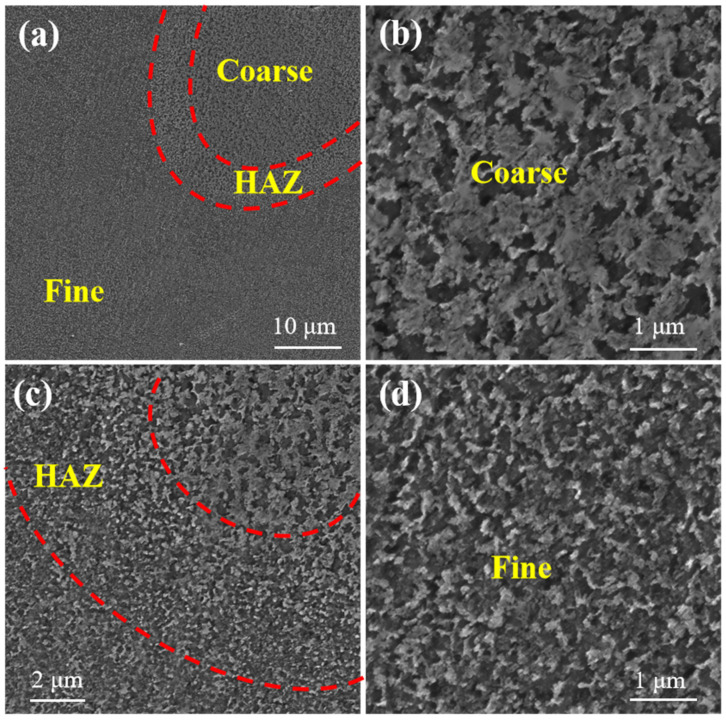
SEM of molten pool boundary: (**a**,**c**) partition of molten pool boundary; (**b**) coarse cellular zone; (**d**) fine cellular zone.

Figure 11 shows the fracture surface of LPBF AlSi10Mg, α-SiC/AlSi10Mg, and β-SiC/AlSi10Mg. As shown in Figure 11a,e,i, the LPBF AlSi10Mg and β-SiC/AlSi10Mg specimens appear relatively dense from the fracture defects. However, pores are clearly visible in the fracture of the LPBF α-SiC/AlSi10Mg specimen, as indicated by the red circles. It can be confirmed that the addition of α-SiC results in poor powder flowability and poor powder spreading quality due to its irregular shape. A few regularly shaped round holes with smooth inner surfaces were identified on the fracture surfaces, as indicated by the yellow arrows in Figure 11c,f,k. These holes are gas pores due to the rapid cooling of the molten pool and residual high-pressure steam; stress concentration is likely to occur around these pores. Thus, there are many porosity defects on the tensile fracture surface. As shown in Figure 11b,f,j, the fracture is distributed at the boundary of the molten pool as determined from the blue arc of the fracture surface morphology, so it can be determined that the mechanical properties of the molten pool boundary are poor. The reason for the poor performance at the boundary is due to the LPBF process. Firstly, Marangoni convection and accompanying liquid capillary forces push the SiC particles; therefore, the SiC particles are mainly located at the boundary of the molten pool. Secondly, the molten pool boundary can be clearly divided into three regions as shown in Figure 12a: the coarse cellular zone (Figure 12b), heat-affected zone (Figure 12c), and fine cellular zone (Figure 12d). Si particles in the coarse cellular zone and heat-affected zone are coarse, so the mechanical properties here are often poor. The fracture occurring at the molten pool boundary is smooth and similar to a cleavage fracture surface, so the fracture mode is a quasi-cleavage fracture. In addition, fine and uniform dimples were found on the fracture surface, as shown in Figure 11d, which showed good plastic fracture characteristics. Meanwhile, SiC particle breakage, as indicated by the red parallel line in Figure 11h, indicates that SiC can achieve load transfer, which has a beneficial effect on performance. As shown in Figure 11i, β-SiC appears on the fracture surface with good interface bonding. When the temperature of the Al melt exceeds 1050 °C, the contact angle between SiC and the Al melt is less than 90°, and there is good wettability between SiC and Al [26]. The melting pool temperature is above 1700 °C during the LPBF process, and it can improve the interface bonding strength of SiC/AlSi10Mg.

## 4. Discussion

Table 4 provides a performance comparison of LPBF aluminum alloys under various parameters. It can be seen that there are significant differences in the parameters selected by other researchers. To facilitate analysis, it is common practice to standardize related parameters into laser energy density. The laser energy density calculation formula is

$$\Phi =\frac{P}{\mathrm{VDt}}$$

where P represents laser power in watts (W); V denotes scanning speed in millimeters per second (mm/s); D stands for scanning distance in micrometers (μm); t represents layer thickness in micrometers (μm); and Φ signifies laser energy density in joules per cubic millimeter (J/mm^3^). Figure 11 presents the corresponding relationship between laser energy density and elongation, and the scatter plot corresponding to the performance data is shown in Table 4.

**Figure 13:**
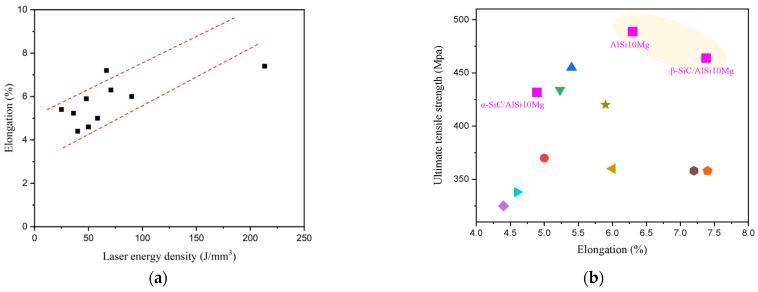
Scatter diagram of the performance and laser energy density [34,35,36,39,40,41,42,43,44]: (**a**) the relationship between laser energy density and elongation; (**b**) the performance data shown in Table 4.

**Figure 14:**
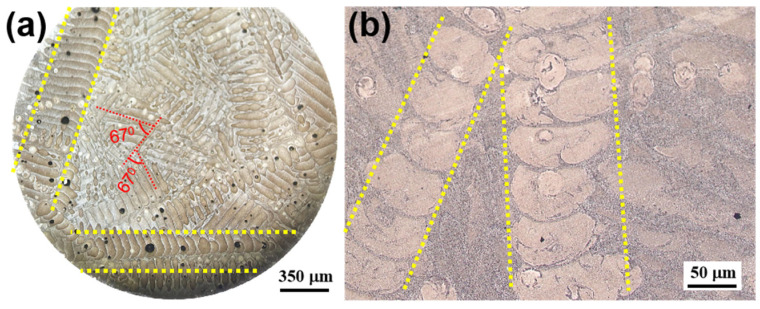
Metallographic photographs of LPBF β-SiC/AlSi10Mg: (**a**) metallographic photograph under a full field-of-eyepiece view; (**b**) metallographic photographs of LPBF β-SiC/AlSi10Mg.

As shown in Figure 13a, the black squares represent the elongation in Table 4, and it is clear that the elongation rises with the increase in laser energy density as shown by dotted red lines. Therefore, the plasticity of the LPBF AlSi10Mg alloy can be improved by appropriately increasing the laser energy density. Significantly, the LPBF AlSi10Mg and LPBF β-SiC/AlSi10Mg specimens in this study exhibit high tensile strength and excellent plasticity, as shown in Figure 13b, which can be analyzed from the following three perspectives:

(1) Most of the studies mentioned in Table 4 adopted uni-directional and bi-directional scanning strategies with a certain rotation angle between layers. Both of these scanning strategies have a longer scanning vector, which can generate excessive accumulated stress and has a negative impact on the performance of the parts. We applied the chessboard scanning strategy, which is achieved by dividing the area into small square cells, reducing the scan vector length and thermal stress [45].
(2) The laser energy density used in this article is relatively high. As analyzed above, a higher laser energy density is better for plasticity.
(3) Laser remelting after the formation of each layer generally improves densification and reduces surface roughness and defects [46,47,48]. In a chessboard scanning strategy, there are scanning overlap areas between the chessboards, and the heat-affected zone generated by the secondary scanning remelting is annealed. Figure 14a shows a metallographic photograph under a full field-of-eyepiece view. The edge of the chessboard is the remelted zone, where a coarse molten pool is formed in a similar manner to the skeleton, as shown in the parallel line area in Figure 14a,b. Due to the good plasticity of the remelted zone, these interconnected plastic skeletons greatly increase the plastic deformation capacity of the composites. Therefore, it is possible to significantly increase the plasticity of the composites while ensuring their strength, and matching the strength and plasticity can be achieved through LPBF process parameter optimization.

## 5. Conclusions

In this study, irregular α-SiC and spherical-like β-SiC were added as reinforcing phases into AlSi10Mg powders. LPBF process parameters were systematically optimized and designed using the BBD of RSM. The effects on the formability and microstructures of the composites caused by the SiC particles with different morphologies were comparatively analyzed. The matching of strength and plasticity was realized by optimizing the process parameters and selecting β-SiC as the reinforcing phase. The main conclusions are as follows:

(1) LPBF AlSi10Mg exhibits excellent plasticity and high tensile strength under the optimized parameters, with an elongation of 7.38% and tensile strength of 463 MPa. LPBF α-SiC/AlSi10Mg shows a decrease in both tensile strength and elongation due to its poor powder flowability and poor powder spreading quality. The tensile strength of LPBF β-SiC/AlSi10Mg is higher than that of LPBF α-SiC/AlSi10Mg. SiC particles can increase the temperature of melt pools and the bonding strength between adjacent molten pools. The metallurgical reaction between SiC and the Al matrix is conducive to the formation of a good bonding interface. The mismatch of the thermal expansion coefficient between the Al matrix and SiC particles leads to geometric dislocations within the matrix, and it can enhance the strength of the composites.
(2) Multiple fracture modes occur when LPBF SiC/AlSi10Mg fails, mainly a quasi-cleavage fracture at the molten pool boundary. Marangoni convection and accompanying liquid capillary forces push the SiC particles located at the boundary of the molten pool. The molten pool boundary can be clearly divided into three regions: the fine cellular zone, heat-affected zone, and coarse cellular zone. The mechanical properties in the coarse cellular zone and heat-affected zone are often poor, so cracks always propagate along the boundary of the molten pool during failure.
(3) The chessboard scanning strategy can form a coarse remelted connected skeleton inside the material, which is of great significance for improving plasticity.

## Figures and Tables

**Table 1:** Chemical composition of AlSi10Mg powders.

Elements	Al	Si	Mg	Fe	Cu	Mn	Ni	Zn	Ti	Pb	Sn	O
Content /wt.%	Bal.	10.2	0.31	0.06	<0.01	<0.01	<0.01	<0.01	<0.01	<0.03	<0.03	0.04

**Table 2:** The parameters of SLM150D.

Equipment Model	Laser Type	Energy /W	Beam Size /μm	Maximum Molding Size /mm	Maximum Scanning Speed /mm/s
SLM150D	Fiber Laser	500	50~60	200 × 200 × 150	5000

**Table 3:** Variables and levels of BBD.

Variable	Code	Coding Level		
		−1	0	1
Laser power, W	A(P)	200	275	350
Scanning speed, mm/s	B(S)	1000	1750	2500
Hatch spacing, μm	C(D)	50	100	150

Note: P, S, and D represent different impact factors; “−1”, “0”, and “1” represent high, intermediate, and low levels, respectively.

**Table 4:** Processes and properties of LPBF aluminum alloys as reported in the literature.

Number	Materials	Laser Power (W)	Scanning Speed (mm/s)	Layer Thickness (µm)	Hatch Spacing (µm)	Laser Energy Density (J/mm^3^)	Scanning Strategy	Ultimate Tensile Strength (MPa)	Elongation (%)	Reference
1	AlSi10Mg	283	2297	30	116	70.81	Chessboard	488.84	6.30	This study
2	2 wt.% α-SiC/AlSi10Mg	283	2297	30	116	70.81	Chessboard	431.75	4.89	This study
3	2 wt.% β-SiC/AlSi10Mg	283	2297	30	116	70.81	Chessboard	463.89	7.38	This study
4	AlSi10Mg	175	1025	30	97.5	58.37	90° rotation	370	5	[34]
5	AlSi10Mg	350	1170	50	240	24.93	90° rotation	455	5.4	[35]
6	AlSi10Mg	350	1140	50	170	36.12	-	434	5.23	[36]
7	Al12Si	320	1455	50	110	39.99	73° rotation	325	4.4	[39]
8	AlSi10Mg	180	1000	40	50	90	Random rotation	360	6	[40]
9	AlSi10Mg	370	1300	30	190	49.93	67° rotation	338	4.6	[41]
10	AlSi10Mg	400	1000	30	200	66.67	-	358	7.2	[42]
11	AlSi10Mg	240	500	50	200	48	-	420	5.9	[43]
12	AlSi10Mg	400	1000	25	75	213.33	67° rotation	358	7.4	[44]

## Data Availability

Data are contained within the article.
